# A large sialolith on the parenchyma of the submandibular gland: A case report

**DOI:** 10.3892/etm.2014.1730

**Published:** 2014-05-26

**Authors:** JAE-HOON JUNG, SUNG OK HONG, KWANTAE NOH, DEOK-WON LEE

**Affiliations:** Department of Oral and Maxillofacial Surgery, Kyung Hee University Dental Hospital at Gangdong, Kyung Hee University, Gangdong-gu, Seoul 134-727, Republic of Korea

**Keywords:** sialolithiasis, salivary stone, submandibular gland, sialoadenectomy

## Abstract

A 45-year-old female was referred to the Department of Oral and Maxillofacial Surgery with the complaint of pain in the right submandibular region and a dry mouth, which had started one week previously. A clinical examination revealed a swelling and tenderness in the right submandibular region. Panoramic radiography and computed tomography identified a sialolith in the submandibular gland. Surgery on the sialolith was subsequently completed under general anesthesia extraorally. A brownish stone was present in the parenchyma of the submandibular gland, measuring 14×10 mm.

## Introduction

Sialolithiasis is the most common disease of the major salivary glands and accounts for ~30% of all salivary disorders. Between 0.01 and 1.0% of the world population is believed to be affected by the disease (1), and the incidence is higher among males aged between 30 and 60 years. The most common location is the submandibular gland, the duct being more frequently affected than the parenchyma (2,3). Sialolithiasis is characterized by obstruction of the salivary secretion by a calculus. This is associated with pain and inflammation and in some occasions with an infection of the affected gland. In a few cases, when the sialolith is small and located near the orifice of the duct, it may be removed following a widening of the orifice with a lacrimal probe. Intraglandular sialoliths require submandibular sialadenectomy or partial parotidectomy. Clinical, radiographic findings are important in determination of the precise location and size in order to indicate the right treatment for the individual patient.

A case is described here which is of interest because the large sized salivary stone is rarely located in the parenchyma of submandibular glands.

## Case report

**Figure 1:**
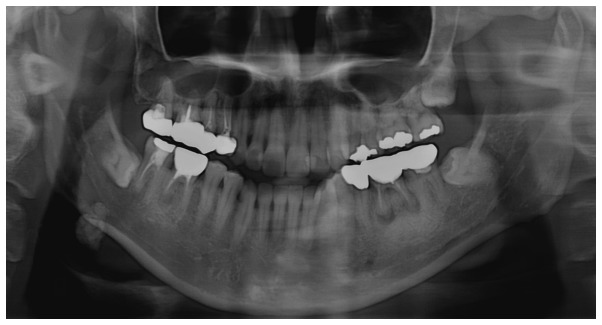
Preoperative panoramic radiography illustrating a radiopaque lesion on the right submandibular area.

**Figure 2:**
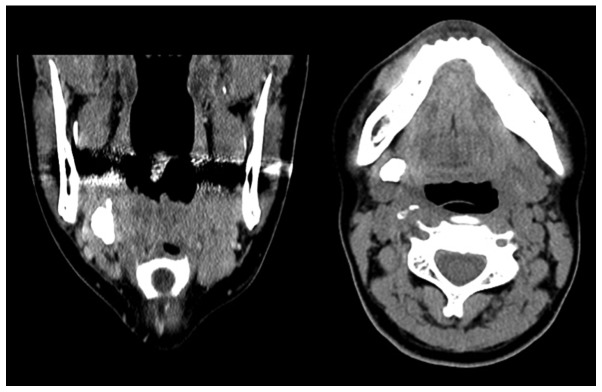
Preoperative contrast-enhanced computed tomography showing the salivary stone on the right submandibular gland.

A 45-year-old female who had no significant medical history was referred to the Department of Oral and Maxillofacial Surgery (Kyung Hee University Dental Hospital at Gangdong, Seoul, Korea) with the chief complaints of pain in the right submandibular region and a dry mouth, which had started one week previously. Clinical examination revealed a swelling and tenderness in the right submandibular region. Panoramic radiography showed a radiopaque mass in the submandibular area (Fig. 1). A few days later, computed tomography was performed, revealing the stone in the right submandibular gland (Fig. 2). We diagnosed this as sialolithiasis on the right submandibular gland.

**Figure 3:**
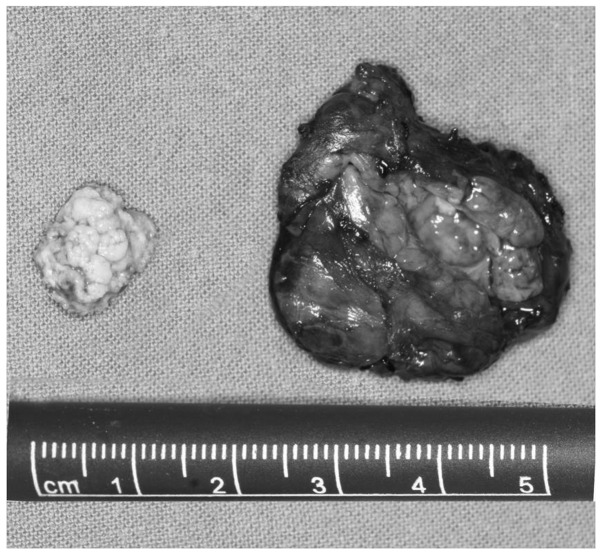
Enucleated submandibular gland and salivary stone in the parenchyma of the submandibular gland.

Surgery to treat the sialolithiasis was subsequently completed extraorally under general anesthesia, due to its location. During surgery, sialoadenectomy was performed around the muscle and the branch of the facial nerve. The main specimen enucleated was an excised submandibular gland, measuring 32×25 mm. The brownish stone was present in the parenchyma of the submandibular gland, measuring 14×10 mm (Fig. 3).

**Figure 4:**
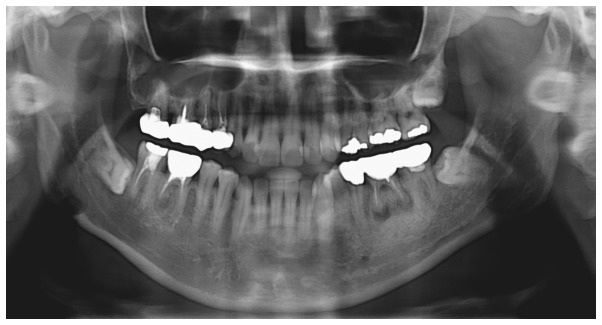
Postoperative panorama showing elimination of the salivary stone on the right submandibular gland.

The postoperative follow-up radiograph showed favorable removal of the salivary stone (Fig. 4). The symptoms and pain of the patient disappeared following surgery and complete healing of the surgical site was observed approximately three weeks subsequently. Informed consent was obtained from the patient and the pateint’s family.

## Discussion

Sialolithiasis is a frequently occurring disease of the salivary glands that is characterized by the obstruction of salivary secretion by a calculus. This is associated with pain and inflammation and, in certain cases, infection of the affected gland may also be present (4). Swelling is the most common symptom, followed by pain, fever and pus secretion (5).

The size of salivary calculi varies from small particles, described as salivary sand, to large concrement formations. The average size of the salivary calculi lies between 3.3 and 17.9 mm (6). According to Lutsmann *et al* (7), sialoliths measuring <10 mm account for 78.8% of cases, while those measuring 10–15 and >15 mm account for 13.6 and 7.65% of cases, respectively. In the present study, the sialolith measured 14×10 mm in size. Salivary calculi are usually located in the ducts of glands and in the hilus, whereas sialoliths on the parenchyma occur in 9–17% of all cases, as stated in previous studies (1,7,8).

The treatment of choice for sialolithiasis is the removal of the obstructing stone by an intraoral approach. In certain instances this method may also be applied for sialoliths located in the hilus of the submandibular gland. In a few cases, when the sialolith is small and located near the orifice of the duct, the sialolith may be removed following a widening of the orifice with a lacrimal probe. Intraglandular sialoliths require submandibular sialoadenectomy or partial parotidectomy (7,9). In the present case, submandibular sialoadenectomy was performed, as the sialolith was large and located in the parenchyma of the gland.

The patient in the present case presented with typical symptoms, and the findings of clinical and radiographic examination were also typical. The diagnosis was made by clinical and radiographic examination, and sialoadenectomy was selected as the treatment method due to the location of the stone. The presented case illustrates the requirement for proper diagnosis and treatment of choice in cases of salivary gland disease. Postoperative follow-up is essential to ensure the patient is symptom- and stone-free in the long-term.

In conclusion, clinical and radiographic findings are important in determining the precise location and size of the sialolith in order to indicate the right treatment for the individual patient.
